# Defining Dysbiosis for a Cluster of Chronic Diseases

**DOI:** 10.1038/s41598-019-49452-y

**Published:** 2019-09-09

**Authors:** Lamont J. Wilkins, Manoj Monga, Aaron W. Miller

**Affiliations:** 1 Lerner College of Medicine, Cleveland Clinic, Cleveland, OH USA; 2 Glickman Urological and Kidney Institute, Cleveland Clinic, Cleveland, OH USA; 3 Department of Inflammation and Immunity, Lerner Research Institute, Cleveland Clinic, Cleveland, OH USA

**Keywords:** Metagenomics, Microbial ecology

## Abstract

The prevalence of many chronic diseases has increased over the last decades. It has been postulated that dysbiosis driven by environmental factors such as antibiotic use is shifting the microbiome in ways that increase inflammation and the onset of chronic disease. Dysbiosis can be defined through the loss or gain of bacteria that either promote health or disease, respectively. Here we use multiple independent datasets to determine the nature of dysbiosis for a cluster of chronic diseases that includes urinary stone disease (USD), obesity, diabetes, cardiovascular disease, and kidney disease, which often exist as co-morbidities. For all disease states, individuals exhibited a statistically significant association with antibiotics in the last year compared to healthy counterparts. There was also a statistically significant association between antibiotic use and gut microbiota composition. Furthermore, each disease state was associated with a loss of microbial diversity in the gut. Three genera, *Bacteroides, Prevotella*, and *Ruminococcus*, were the most common dysbiotic taxa in terms of being enriched or depleted in disease populations and was driven in part by the diversity of operational taxonomic units (OTUs) within these genera. Results of the cross-sectional analysis suggest that antibiotic-driven loss of microbial diversity may increase the risk for chronic disease. However, longitudinal studies are needed to confirm the causative effect of diversity loss for chronic disease risk.

## Introduction

Many chronic diseases are on a trend of increasing prevalence. Cardiovascular disease, obesity, diabetes, urinary stone disease (USD), asthma, and inflammatory bowel disease (IBD) are all on the rise, among others^1–6^. These chronic inflammatory diseases often exist as co-morbidities with common physiological manifestations, which both compound the burden on patients and suggests common origins^7–9^. While a number of genetic and environmental factors contribute to the manifestation of chronic disease, an emerging hypothesis postulates that dysbiosis, an imbalance in the composition and metabolic capacity of our microbiota, increases the risk of developing chronic disease^10–13^. Dysbiosis can contribute to the onset of chronic disease in one of three general ways. First, pathogens and their functions can be acquired or opportunistically overgrow to promote disease, termed gain of function dysbiosis. Gain of function dysbiosis leads to infectious diseases such as cholera or streptococcal pharyngitis, but can also lead to chronic inflammation^14–16^. Second, health-protective bacteria and their functions may be lost or suppressed, which then promotes the onset of disease, termed loss of function dysbiosis. Loss of function dysbiosis has been linked to chronic diseases such as IBD, USD, obesity, and others^17–26^. Finally, some combination of loss and gain of function dysbiosis may be required for the onset of disease, such as with recurrent *Clostridium difficile* infection^27^.

The complexity of the microbiome presents unique challenges to our understanding of the role of dysbiosis in disease. Each individual harbors thousands of unique microbial operational taxonomic units (OTUs)^28,29^. Compounding this issue is the considerable inter-individual variability in the composition of the microbiome^30,31^. Given the hyper-variable nature of high-throughput 16S rRNA data combined with the high levels of inter-individual variability, any two random populations of individuals are likely to harbor a proportion of OTUs unique to each population, with no relevance to any phenotype. To help restrict false-discoveries associated with this issue, false-discovery rate corrections are commonly integrated into microbiome studies^32^. Despite the limitations of microbiome studies, many clinical studies use the differential abundance of OTUs between healthy and disease populations at a single time-point to determine if dysbiosis contributes to the disease, which can lead to erroneous results when it comes to the nature of dysbiosis and microbial taxa involved. Thus, a number of questions remain surrounding conclusions that dysbiosis contributes to the manifestation of disease. First, is the differential abundance of OTUs between two populations driven by inter-individual variability of the two populations or disease status? Second, what features of microbial taxa contribute to them being identified as dysbiotic? Finally, are there taxa consistently found to be dysbiotic across chronic disease states, indicative of common, dysbiosis-driven disease processes?

To address these questions of defining dysbiosis, we performed an independent analysis of microbiome data from multiple sources that spanned a cluster of chronic diseases that included USD, cardiovascular disease, obesity, diabetes, and kidney disease. Specifically, we quantified the use of antibiotics in disease populations compared to healthy controls, which can lead to a loss in microbial diversity relevant to disease processes^33–40^, quantified metrics of loss or gain of microbiota diversity associated with each disease state relative to stochastically defined populations, determined the similarities and differences in microbial taxa enriched and depleted in disease populations, and determined which of these taxa were differentially abundant more than expected given their genus-level diversity in samples. These diseases often exist as comorbidities in patients and have all been linked to the microbiome to some degree. We hypothesize that if the diseases in question are driven in part by dysbiosis, that there will be common metrics of dysbiosis that include antibiotic use, loss or gain of microbial diversity, and microbial taxa associated with disease.

## Results

### Datasets

For cardiovascular disease, obesity, diabetes, kidney disease, and healthy counterparts, 16S rRNA sequences from the stool and associated sample metadata were drawn from the April 26^th^, 2017 version of American Gut Project data (AGP), which has an extensive list of metadata that includes antibiotic history and the presence or absence of several disease states^41^. Samples for sequencing were collected between December 2012 and April 2017 from individuals from a global population. Healthy individuals were defined by the “I do not have this condition” entry for diabetes, cardiovascular disease, and kidney disease, as well as “normal” for body mass index (“BMI_CAT” in the metadata). Those with “diagnosed” for cardiovascular disease, diabetes, or kidney disease, or with “obese” for BMI_CAT were assigned to the appropriate chronic disease state. Health status for each defined term were based on self-reported medical diagnoses which may bias results^41^. A random number generator was used to assign random numbers to each sample that fit one of the defined health states for the AGP data and the samples with the lowest 300 randomly assigned numbers for each health status were extracted for further analysis, except for kidney disease which only had 111 entries. For kidney disease, we extracted samples with the lowest 100 numbers for further analysis. For USD, which is not represented in the AGP metadata, 16S rRNA sequences and associated sample metadata were downloaded from all publicly available metagenomic data from clinical USD studies published at the time of analysis^18,20,42,43^. Samples for USD studies were collected between 2015–2019 and originated in the United States, Canada, India, or China. Samples were defined as healthy or USD based on the criteria used in the original study. The entire combined dataset spanned a total of 1468 samples (Table 1).

**Table 1:** Characteristics of datasets used in the meta-analysis.

Disease State	No. of Samples	Sample Type	Source
Healthy	300	Stool	AGP
Cardiovascular disease	300	Stool	AGP
Obesity	300	Stool	AGP
Diabetes	300	Stool	AGP
Kidney disease	100	Stool	AGP
Healthy	18	Stool	ref.^36^
USD	18	Stool	ref.^36^
Healthy	13	Stool	ref.^37^
USD	13	Stool	ref.^37^
Healthy	15	Stool	ref.^18^
USD	24	Stool	ref.^18^
Healthy	43	Stool	ref.^20^
USD	24	Stool	ref.^20^

### Association of antibiotics and chronic disease

**Figure 1:**
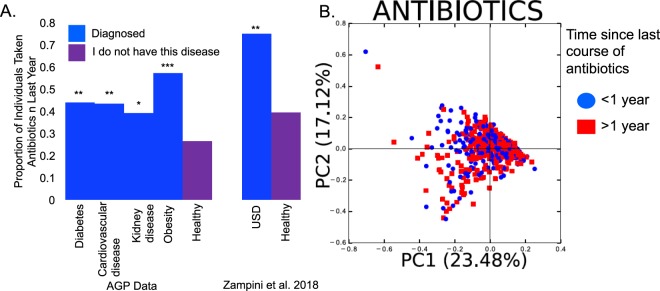
The effect of antibiotics on chronic disease and the microbiota. (**A**) Antibiotic use within the last year for individuals with or without chronic disease. For diabetes, cardiovascular disease, kidney disease, obesity, and their healthy counterparts, antibiotic history was derived from the subset of AGP samples randomly selected for this study (N = 300 for each group except kidney disease which had 100 samples; Table 1). Only one study on the microbiome of USD patients included metadata associated with antibiotic use (N = 43 healthy individuals and 24 individuals with USD; Table 1). Proportions of antibiotic use were compared between chronic disease states and healthy populations with a relative risk ratio followed by a post-hoc Fisher’s exact test, which was Holm’s corrected for multiple comparisons. *p < 0.05; **p < 0.01; ***p < 0.001 compared to the healthy population. (**B**) PCoA plot based on a weighted UniFrac analysis the microbiome composition from the AGP data. Community composition based on antibiotic use was compared by PERMANOVA with 999 permutations. **p** = **0.006**.

For all chronic disease states, individuals were significantly more likely to have taken antibiotics in the last year compared to healthy individuals (Fig. 1A). Furthermore, antibiotic use in the last year was significantly associated with microbiota composition as assessed by a weighted UniFrac analysis followed by a post-hoc PERMANOVA of the regularized AGP datasets (Fig. 1B)^20,44^.

### Nature of dysbiosis in chronic disease

**Figure 2:**
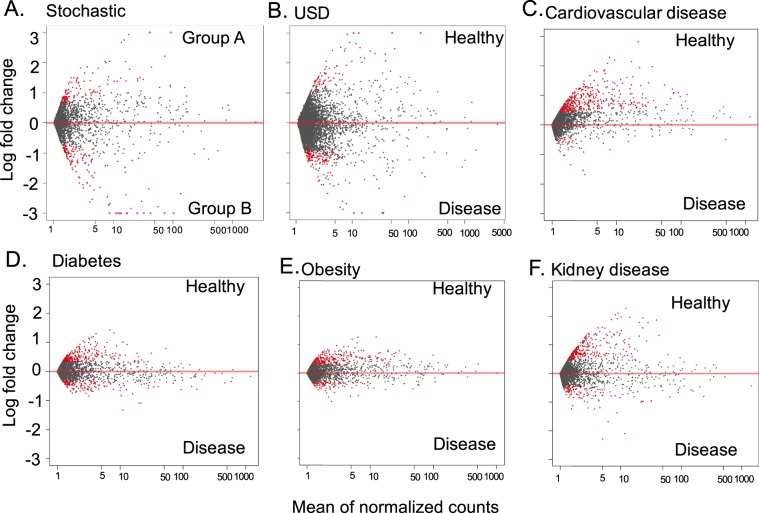
An example of differential abundance analysis for each of the disease states. Each dot represents an OTU. Grey dots are OTUs that do not exhibit significant differential abundance, while red dots are differentially abundant OTUs. (**A**) Stochastic metadata; (**B**) USD; (**C**) Cardiovascular disease; (**D**) Diabetes; (**E**) Obesity; (**F**) Kidney disease.

**Figure 3:**
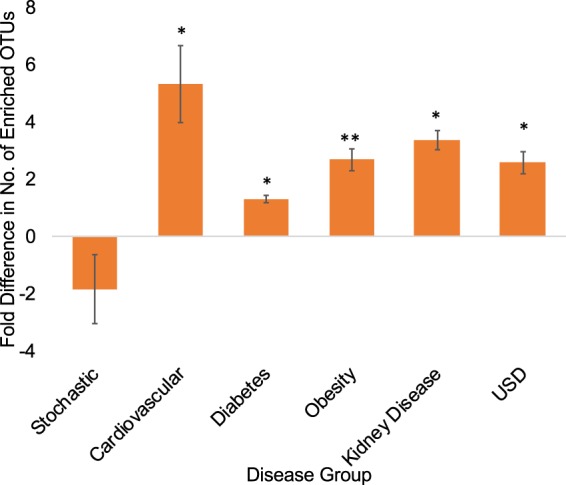
Average fold difference in the number of OTUs enriched in either the healthy group/stochastic group 1 or in the disease group/stochastic group 2. Positive values reflect greater enrichment in healthy group/stochastic group 1, whereas negative values reflect greater enrichment in disease group/stochastic group 2. Significance was determined with a one-sample t-test against an expected value of 1. *p < 0.05; **p < 0.01; ***p < 0.001.

The differential abundance of OTUs between healthy and disease populations was used to determine if each of the disease states were associated with a depletion or enrichment of microbial diversity compared to healthy controls, with the number of OTUs enriched or depleted in disease quantified for each pairwise comparison (Fig. 2). The average fold difference in OTUs enriched in healthy cohorts vs. disease cohorts reveals that for each disease state, there was significantly more OTUs enriched in healthy cohorts than disease cohorts, indicative of a loss of microbial diversity in the gut (Fig. 3). No significant bias for group 1 or 2 was detected when health status was stochastically assigned to samples through random assignment (see methods).

### Strength of dysbiotic taxa and commonalities across chronic disease states

**Figure 4:**
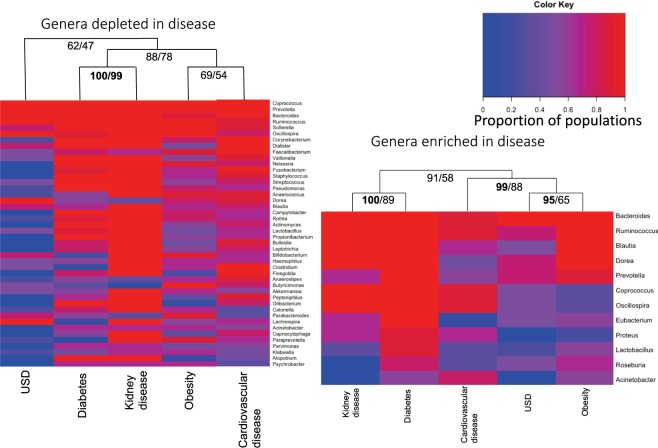
Heatmaps showing the most common dysbiotic genera for each disease. Genera were counted for each independent population comparison had at least one dysbiotic OTU associated with it. The proportion of populations each genera showed up in is plotted. (**A**) Genera depleted in the disease populations (potential probiotic bacteria lost); (**B**) Genera enriched in disease populations (potential pathogenic bacteria). Hierarchal cluster analysis shows clustering of disease states with the approximately unbiased alpha levels (AU) and bootstrap probability (BP) provided for each cluster (AU/BP). AU values > 95 are considered significant and are bolded.

The proportion of independent healthy x disease comparisons in which a particular genus had at least one OTU that was differentially abundant was plotted on a heatmap (Fig. 4). The heatmap reveals that the most common dysbiotic genera were the *Coprococcus, Prevotella*, and *Bacteroides* for OTUs enriched in the healthy cohorts. For OTUs enriched in disease cohorts, the *Bacteroides, Ruminococcus*, and *Blautia* genera were most common. Hierarchal clustering reveals statistically significant similarities between diabetes and kidney disease when considering potential health protective bacteria lost. When considering potential pathogenic bacteria acquired, obesity and USD exhibit a statistically significant cluster, which also clusters with cardiovascular disease. Diabetes again clustered with kidney disease with statistical significance. Additionally, from the heatmaps, it is apparent that each of the diseases is associated with a loss of diverse genera more so than the gain of microbial genera, largely corroborating analyses based on antibiotic use and the number of dysbiotic OTUs (Fig. 4).

### Impact of genus diversity on dysbiotic OTUs

**Figure 5:**
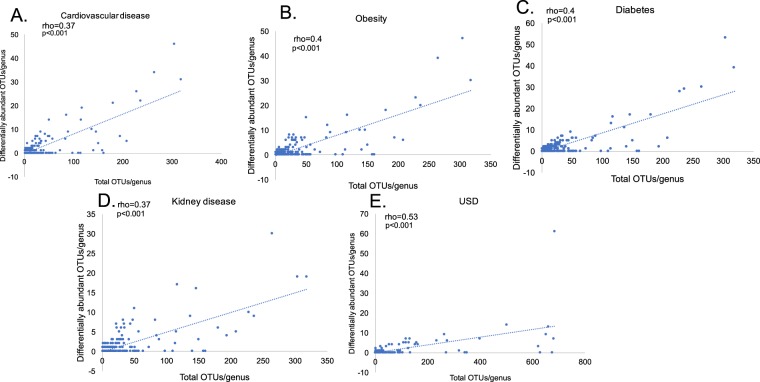
Total genus diversity vs. dysbiotic OTUs per genus. Correlations were calculated with a Spearman’s rank order correlation (r). (**A**) Cardiovascular disease; (**B**) Obesity; (**C**) Diabetes; (**D**) Kidney disease; (**E**) USD.

When the total number of OTUs identified in each genus is plotted against the number of dysbiotic OTUs identified in each genus, there is a significant correlation between the two factors, reflecting the fact that dysbiosis is driven in part by the number of constituent OTUs in each genus (Fig. 5). However, the number of genera determined to be dysbiotic more than expected given genus level diversity ranged from one (USD) to 14 (kidney disease). Of these, the *Bacteroides* genus was more likely to be dysbiotic for all diseases and *Corynebacterium* for all but USD. The *Anaerococcus* genus was more likely to be dysbiotic than expected for three of the five disease states (Table 2).

**Table 2:** Microbial genera that were identified as dysbiotic more than expected based on the number of unique OTUs identified per genus.

Genus	USD	Diabetes	Kidney Disease	Obesity	Cardiovascular disease
Bacteroides	*	*	*	*	*
Corynebacterium		*	*	*	*
Anaerococcus			*	*	*
Coprococcus				*	*
Oscillospira		*			*
Prevotella			*		*
Rothia			*	*	
Sutterella			*		*
Bifidobacterium					*
Blautia		*			
Eubacterium			*		
Faecalibacterium					*
Fusobacterium			*		
Leptotrichia			*		
Parabacteroides			*		
Peptoniphilus			*		
Porphyromonas			*		
Ruminococcus		*			
Staphylococcus			*		
Veillonella			*		

## Discussion

In the last ten years, there has been an explosion of metagenome-wide association studies (MWAS) that seek to determine if the microbiome contributes to disease processes. Importantly, these studies have revealed that there is both considerable diversity in the microbiome with hundreds to thousands of unique bacterial species in the gut alone^28,29^ and a high amount of inter-individual variability^30,31^. Given these unique characteristics of the microbiome, studies on any one population of individuals can be driven simply by the individual variability and are thus unreliable. Despite these limitations, one leading hypothesis has emerged that postulates that the loss of diversity from our microbiome is increasing the risk of developing chronic disease^45–47^. Several questions remain about defining the nature of dysbiosis for any one particular disease. First, are MWAS results driven by inter-individual variability and study design, or are there consistent responses by disease? Second, are different diseases consistently associated with the loss of beneficial bacteria or the gain of harmful bacteria? Finally, is dysbiosis driven by a general loss or gain of microbial taxa or are there specific taxa that are more important than others? To address these questions, we conducted a meta-analysis of MWAS data from multiple sources for a cluster of disease states that included USD, diabetes, cardiovascular disease, obesity, and kidney disease.

Some of the most striking results of the current study is that every disease in the cluster of chronic diseases exhibited a statistically significant association with past year antibiotic use and that antibiotics had a statistically significant association with gut microbiota composition up to a year after use, both of which supports the loss of function dysbiosis hypothesis (Fig. 1). Further evidence that a loss of microbial diversity increases risk of developing the chronic diseases studied here comes from the differential abundance analyses, in which individuals from each of the disease states exhibited a statistically significant propensity for the reduced presence of bacteria in their gut compared to their healthy counterparts (Fig. 3).

Past studies have shown both direct and indirect links between antibiotic use and the risk of developing USD^20,35,48–51^ and obesity^37–39,52,53^. For diabetes, the literature is mixed with some studies concluding that antibiotics increase the risk of diabetes^54–57^, some that conclude repeated antibiotic use but not single antibiotic use increases diabetes risk^58^, and some that conclude antibiotics protect against diabetes^54,59,60^. For cardiovascular disease, animal studies show that antibiotics lead to a reduction in microbial activity that promotes cardiovascular disease^61^. However, outcomes using antibiotic therapy to treat cardiovascular disease in a clinical setting to date have been mixed^62,63^. In addition to the specific diseases above, another meta-analysis of MWAS studies that spanned multiple unrelated diseases has been published. In that meta-analysis, it was found that some diseases such as enteric diarrheal disease, *Clostridium difficile* infection, and IBD were associated with loss of function dysbiosis, whereas diseases such as colorectal cancer, autism spectrum disorder, liver diseases, and Parkinson’s disease were associated with gain of function dysbiosis^64^.

Among the taxa that were most consistently found to be depleted in disease individuals were the *Bacteroides, Coprococcus, Prevotella, Ruminococcus*, and *Sutterella* (Fig. 5). However, subsequent analysis suggests that the association of some of these taxa with dysbiosis is closely tied to their number of constituent OTUs in the gut, indicative of a non-specific response. Some taxa, primarily *Bacteroides, Corynebacterium*, and *Anaerococcus* were identified as dysbiotic more than expected given their diversity, indicative of a more specific physiological interaction between these taxa and disease (Table 2). Past studies have concluded that *Bacteroides* either has a health-protective^65–67^ or health-antagonistic response^68–70^. Results from the current analysis show that this genus is strongly associated with both health and disease in terms of the number of independent populations it was found to be associated with (Fig. 4) and thus it is likely that some OTUs within the genus provide more of a protective effect and others more of a detrimental health impact. The *Corynebacterium* and *Anaerococcus* genera were only associated with the healthy individuals in our meta-analysis. *Corynebacterium* is most often associated with diphtheria, an illness that primarily affects the respiratory system^71^. The number of independent healthy populations associated with *Corynebacterium* here suggests that this genus of bacteria may play a more beneficial role in the context of the gut microbiota. *Anaerococcus sp*. are commonly associated with the normal flora of the skin, mouth, and gut, but are also often recovered from clinical specimens such as vaginal discharge and chronic infectious wounds^72^.

Numerous MWAS studies have demonstrated statistically significant interactions between the microbiome and disease processes. However, questions remain about the consistency of results obtained from single studies and whether there are common dysbiotic origins for related diseases. Importantly, our results show that for the cluster of chronic diseases that span USD, diabetes, kidney disease, obesity, and cardiovascular disease, there is a statistically significant association with antibiotic use and a loss of microbial diversity. Furthermore, several of the dysbiotic taxa are shared between different disease states suggesting that there are some common dysbiotic associations for these diseases. However, as the current data represents a retrospective analysis of data from a single time-point, prospective, longitudinal clinical studies are required to understand the underlying mechanisms between antibiotics, loss of function dysbiosis, and the onset of chronic disease.

## Materials and Methods

### Data processing

Raw sequencing data for all studies were demultiplexed and quality-controlled with default parameters in QIIME^73^. An open reference strategy was used to assign OTUs with 97% homology compared to a reference database composed of the Greengenes V13.8 dataset^74–76^. Datasets were combined prior to OTU assignment to allow for direct comparisons. OTUs that did not match any of sequences in the reference database were then classified *de novo*, which first clusters unclassified sequences based on homology, picks a representative sequence, and assigns taxonomy based on a 94% similarity threshold to the reference database^77^. Chloroplasts, mitochondria, and chimeric sequences were removed from datasets as well as those that had <10 representations across each dataset as previously described^74,75^. The final OTU count across all datasets was 35,582. Datasets were regularized with a negative binomial Wald test, executed by the DESeq2 algorithm, which fits the data to a negative binomial distribution, to account for differences in sequencing depth in each sample, but does not account for the compositional nature of the data^78,79^.

### Association of antibiotics and chronic disease

To determine if antibiotics were associated with chronic disease, we quantified the proportion of individuals who had taken antibiotics in the last year for cardiovascular disease, obesity, diabetes, and kidney disease compared to their healthy counterparts in the AGP data. For USD, only one of the four studies included in our meta-analysis had metadata associated with antibiotic use^20^. Proportions were compared with a relative risk ratio followed by a post-hoc, Fisher’s exact test, using a Holm’s correction for multiple hypotheses.

In order for antibiotic use to have a meaningful impact on chronic disease, we expect that antibiotics would exhibit a statistically significant association with microbiota composition. Therefore, we also conducted a weighted UniFrac beta-diversity analysis on all regularized data from the AGP dataset, which were all collected and processed by the same group, followed by a post-hoc PERMANOVA against antibiotic use in the past year.

### Defining dysbiosis for each disease state

To determine the nature of dysbiosis for each disease state, while considering the multi-dimensional and variable nature of microbiome data, we divided the samples into the following independent populations. For USD data, each study was considered an independent population and the healthy vs. USD groups were compared against each other within each study. Four populations were available for USD and their healthy counterparts. For AGP data, each category of health states was sub-divided into three independent sets of 100 samples by assigning samples to one of three groups through the lowest, middle, and highest 100 random numbers previously assigned. For kidney disease, only one subset was available. Subdivisions provided three independent populations for AGP healthy individuals, cardiovascular disease, obesity, and diabetes. One population was available for kidney disease. Additionally, random numbers were reassigned to AGP data independent of metadata. The random numbers generated were used to assign three independent populations each for stochastic groups 1 & 2. Groups with stochastic metadata were used to determine if our definition of dysbiosis for each disease state were driven by disease or some other stochastic process.

Following subdivisions, differential abundance analysis, calculated as the log fold-change divided by standard error of negative binomial regularized data (lfcse), was conducted on all relevant pairwise comparisons of independent populations using the DESeq2 algorithm^78^. All p-values for differentially abundant OTUs were false-discovery rate corrected, following the Benjamini-Hochberg method^80^. Differential abundance analysis was chosen to define dysbiosis because it gives the specific OTUs enriched in both healthy and disease populations rather than net differences in diversity or community composition as with alpha- and beta-diversity respectively. Thus, this metric is suitable for identifying situations in which a combination of loss and gain of function dysbiosis may contribute to disease processes. For USD data, since samples and data were collected independently by four different research groups, differential abundance analysis between healthy and USD populations within each study was conducted. For AGP data, since all samples were collected, processed, and sequenced by the same group, each independent healthy population was compared to each disease population. This resulted in nine pairwise comparisons for cardiovascular disease, diabetes, obesity, and stochastic groups 1 & 2, along with three pairwise comparisons for kidney disease.

From the results of independent differential abundance analyses, the fold difference in OTUs between the healthy and disease populations was calculated as the number of OTUs enriched in the healthy group divided by the number of OTUs enriched in the disease group. Absolute values were compared with a one-sample t-test against an expected value of 1, indicative of no difference, and p-values were Holm’s corrected for multiple comparisons. For purposes of plotting fold difference, raw values below 1 were inverted and made negative.

### Strength of dysbiotic taxa and commonalities across chronic disease states

To determine the consistency of dysbiotic taxa identified and if there were common dysbiotic genera across the different chronic diseases, differentially abundant OTUs for each comparison were first reduced to genera, which allows for greater comparison between each disease state but comes with the limitation that physiologically disparate OTUs can exist within the same genus. The OTUs that were not classified at least to the genus level were removed from further analysis. We counted the number of times a microbial genus had at least one OTU that was enriched in either the healthy or disease populations across all pairwise comparisons for each disease. Genera enriched in the healthy populations were considered “potentially beneficial” whereas those enriched in the disease populations were considered “potentially pathogenic”. The proportion of independent analyses that each genus appeared in was plotted in a heatmap in R statistical software using the heatmap.2 function with dendograms generated by the hclust function to show clustering by disease state. The bootstrap probability and approximately unbiased alpha levels were calculated by the pvclust package with 1000 bootstraps^81^.

### Influence of genus diversity on dysbiotic genera

Next, we sought to determine if dysbiosis was driven by a general loss or gain of bacteria in the gut or if some microbial genera had a greater association with disease than expected by the number of constituent OTUs within the genus. To do this, for each disease, we plotted the total number of OTUs detected per genus against the number of dysbiotic OTUs detected per genus and determined if there was a Spearman correlation significantly different than 0 with the cor.test function in R statistical software. Next, genera in which the number of dysbiotic OTUs was greater than three standard deviations above what was expected based on genus diversity were considered to be highly associated with dysbiosis, following the three-sigma rule^82^.

## Data Availability

Sequence reads for USD are available at the Sequence Read Archive under Accession #’s SRP140641, SRP140933, PRJNA382644, PRJNA304689. American gut project data is available at the European Bioinformatics Institute under accession # PRJEB11419.
